# Variations in salinity tolerance of malaria vectors of the *Anopheles subpictus *complex in Sri Lanka and the implications for malaria transmission

**DOI:** 10.1186/1756-3305-4-117

**Published:** 2011-06-24

**Authors:** Sinnathamby N Surendran, Pavillupillai J Jude, Ranjan Ramasamy

**Affiliations:** 1 Department of Zoology, Faculty of Science, University of Jaffna, Jaffna, Sri Lanka; 2 Institute of Health Sciences, Universiti Brunei Darussalam, Gadong, Brunei Darussalam

## Abstract

**Background:**

*Anopheles subpictus sensu lato*, a widespread vector of malaria in Asia, is reportedly composed of four sibling species A-D based on distinct cytogenetic and morphological characteristics. However *An. subpictus *species B specimens in Sri Lanka are termed *An. subpictus *B/ *An. sundaicus *because of recent genetic data. Differences in salinity tolerance and coastal/inland prevalence of *An. subpictus *sibling species that were not previously established in Sri Lanka are presented here.

**Results:**

Specimens with morphological characteristics of all four Indian *An. subpictus *sibling species were found in Sri Lanka. Sibling species A, C and D tended to be predominant in inland, and *An. subpictus *species B/*An. sundaicus*, in coastal localities. Sibling species C was predominant in both adult and larval inland collections. Larvae of *An. subpictus *B/*An. sundaicus *were found in inland and coastal sites, including a lagoon, with salinity varying from 0 to 30 ppt. *An. subpictus *sibling species A, C and D larvae were present in water of salinity between 0 to 4 ppt. *An. subpictus *C, D and *An. subpictus *B/*An. sundaicus *larvae showed compatible differential salinity tolerance in laboratory tests. The first instar larvae of *An. subpictus *B/*An. sundaicus *showed 100% survival up to 15 ppt in comparison to species C and D where the corresponding values were 3 ppt and 6 ppt respectively. However all third instar larvae of *An. subpictus *B/*An. sundaicus *survived up to 30 ppt salinity whereas *An. subpictus *C and D tolerated up to 4 ppt and 8 ppt salinity respectively.

**Conclusions:**

The results suggest that *An. subpictus *species B/*An. sundaicus *breed in fresh, brackish and nearly saline water while *An. subpictus *species C and D do so in fresh and less brackish waters in Sri Lanka, as in India. Because of the established role of *An. sundaicus s.l*. and *An. subpictus s.l*. as malaria vectors, the findings indicate a need for greater monitoring of brackish water breeding habitats in Asia. Tolerance to 15 ppt salinity may also constitute a simple method for differentiating *An. subpictus *B/*An. sundaicus *larvae from those of *An. subpictus *species C and D in field studies.

## Background

The major vector of *Plasmodium falciparum *and *Plasmodium vivax *malaria in Sri Lanka [1,2] is *Anopheles culicifacies *species E, but *Anopheles subpictus s.l *is also a malaria vector in many parts of the island [3-5] and elsewhere in Asia [6-8]. *An. subpictus s.l*. can additionally transmit filarial parasites and Japanese encephalitis and West Nile viruses [6,9,10]. The taxon *An. subpictus *is reported to be a complex of four sibling species, *viz*. A, B, C and D in India [6,7], that are differentiated through characteristic paracentric fixed inversions on the X-chromosome and stage-specific morphometric characteristics e.g. the number of ridges in egg floats, larval mesothoracic seta 4, pupal setae and ornamentation of the palpi of adult females [7]. Previous studies have reported the presence of all four sibling species in Sri Lanka based on the morphological characteristics observed in India [11,12].

Existing evidence suggests that members of the Subpictus Complex are able to breed in inland and coastal habitats, that they are generally zoophagic, both exophilic and endophilic, and that they are able to tolerate a range of salinities in their breeding sites [1,6,12-14]. The limited data on bio-ecological differences between members of the *An. subpictus *complex in Sri Lanka and elsewhere in Asia and their geographical distribution have been recently reviewed [1,6,13]. They indicate a need for more detailed studies to establish differences between sibling species that contribute to the range of bio-ecological characteristics observed in the Subpictus Complex.

*An. subpictus *species B is generally found in coastal areas of India and is reported to show greater salinity tolerance when compared to species A, C and D in breeding sites in India [14] and elsewhere in Asia [13]. Many *An. subpictus *species B specimens from Eastern Sri Lanka, and some others from Southeast Asia, although morphologically similar to *An. subpictus*, are genetically closer to *Anopheles sundaicus *[15]. Therefore mosquitoes in Sri Lanka with morphological characters of Indian *An. subpictus *B are termed *An. subpictus *B/*An. sundaicus *in this article. The differential salinity tolerance and preferences for coastal or inland breeding sites of individual members of the Subpictus Complex are yet to be clearly established in Sri Lanka. Characterization of differences in their larval habitats is important as there are known genetic differences between corresponding anophelines in Sri Lanka and neighbouring India e.g. *An. culicifacies *species B and E [16].

Differences in bio-ecological traits of sibling species of malaria vectors are essential for adopting appropriate vector control measures. Characteristics of malaria transmission in coastal areas in the North Central Province [12] and inland areas of North Central and Eastern Provinces [3-5] of Sri Lanka indicate the involvement of *An. subpictus *sibling species B and species C/D/A in the respective areas. Larval control measures have hitherto been almost exclusively applied to fresh water breeding sites of malaria vectors in Sri Lanka. The salinity tolerance and prevalence in coastal and inland sites of mosquitoes identified morphologically as belonging to the *An. subpictus *complex in sites located in the Eastern and Northwestern provinces of Sri Lanka were therefore investigated and the results reported here.

## Results

### Field collections of blood-fed adult *An. subpictus *females of different sibling species

**Figure 1:**
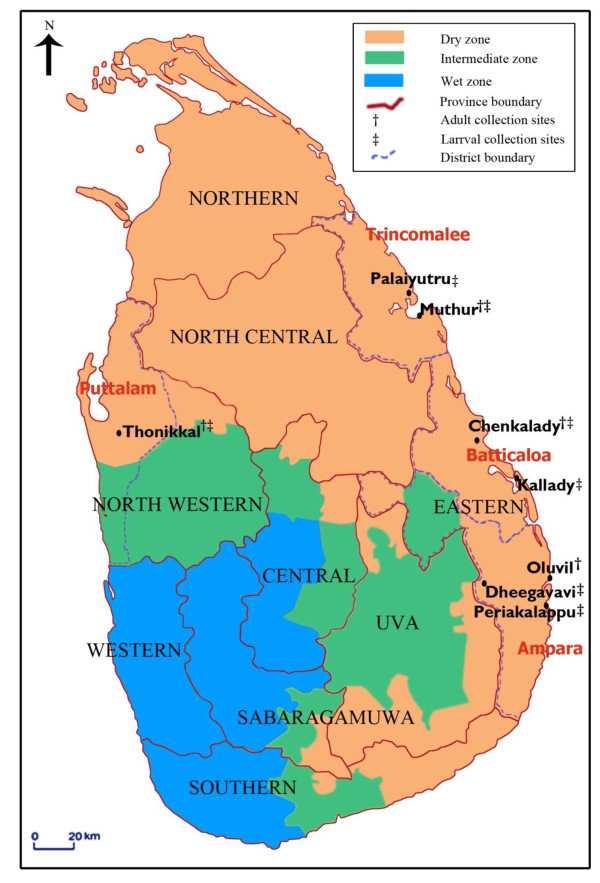
**Map showing the adult and larval collection sites and the climatic zones (dry, intermediate and wet) of Sri Lanka**.

Blood-fed *An. subpictus*-like females could be collected from sites in all the four Sri Lankan districts studied [Figure 1]. Of the 4098 blood-fed adult females that were collected during the study, 1247 laid eggs. Microscopic determination of the numbers of ridges in egg floats laid by them suggested the presence of *An. subpictus *B/*An. sundaicus *and *An. subpictus *species A, C and D [Table 1]. While sibling species A, C and D tended to predominate in inland localities (localities located ≥2.5 km from the coast), species B/*An. sundaicus *tended to be more prevalent in coastal areas. Among the inland species, sibling species C was predominant in both adult and larval collections [Tables 1 and 2]. There were fewer *An. subpictus *A-like females compared to other sibling species in the collections.

**Table 1:** Adult females in field collections characterized as *An.subpictus *sibling species A, B/*An. sundaicus*, C and D through morphology of laid eggs

Districts	Location	Type of locality	Numbers of adult females			
			
			A	B/*An. sundaicus*	C	D
Batticaloa	Chenkalady	Inland	4	187	387	82
Ampara	Oluvil	Coastal	0	65	67	20
Puttalam	Thonikkal	Inland	0	74	120	34
Trincomalee	Muthur	Coastal	0	24	0	0
		Inland	0	33	128	22

**TOTAL**			**4**	**383**	**702**	**158**

**Table 2:** Larvae with characteristics of *An.subpictus *sibling species A, B/*An. sundaicus*, C and D collected from different sites during the period February 2009 - June 2010

District	Location	Type of locality	Type of breeding site	Salinity (ppt)	Number of larvae of the *An. subpictus *sibling species			
					
					A	B	C	D
Batticaloa	Chenkalady	Inland	Pond	0	-	06	26	09
	Kallady	Coastal	Sand pool	0	24	-	54	81
			Sand pool	2	7	215	147	32
			Sand pool	4	-	253	27	07
	Kallady	Coastal	Sand pool	0	-	-	12	-
			Sand pool	3	-	-	19	3
			Lagoon margin	22	-	68	-	-
			Lagoon margin	30	-	112	-	-

Puttalam	Thonikkal	Inland	Quarry	0	-	67	114	-

Ampara	Periakalappu	Coastal	Sand pool	18	-	12	-	-
			Sand pool	30	-	5	-	-
	Dheegavavi	Inland	Pond	0	-	24	93	49

Trincomalee	Muthur	Coastal	Sand pool	0	31	18	25	-
			Sand pool	2	-	41	06	-
	Muthur	Coastal	Well	0	-	55	87	-
		Coastal	Sand pool	2	-	12	27	-
	Palaiyutru	Inland	Irrigation canal	0	7	26	103	26

### Characteristics of larval breeding habitats of *An. subpictus *sibling species

**Figure 2:**
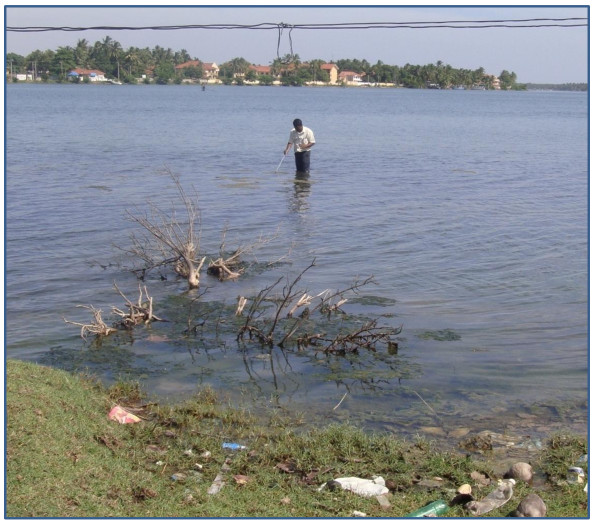
**Photograph of the Kallady lagoon site (30 ppt salinity) where *An. subpictus *species B*/An. sundaicus *larvae were collected in association with marine algae >15 m into the lagoon**.

In parallel studies, 1930 larvae could be classified by examination of seta 4M and the results showed that larvae with characteristics of all four *An. subpictus *sibling species were present in both inland and coastal locations [Table 2]. However there was a tendency for larvae with characteristics of *An. subpictus *B/*An. sundaicus *to predominate in coastal localities [Table 2]. Larvae with characteristics of *An. subpictus *B/*An. sundaicus *were collected from both inland and coastal locations with salinity levels varying from 0 to 30 ppt (fresh, brackish and saline water are defined as having <0.5, 0.5 to 30 and >30 ppt salt respectively). Furthermore they were the only larvae collected from sites where the salinity was >4 ppt. Larvae with characteristics of *An. subpictus *A, C and D were only collected from sites where the salinity was ≤ 4ppt. Collection of large numbers of *An. subpictus *A, C and D larvae in coastal areas was associated with the rainy season (October to January) during which the salinity levels of the water bodies decreased due to dilution by rain water [Table 2]. All the breeding sites, except that in Muthur, were exposed to direct sunlight. All sites, except those in Palaiyutru and Thonikkal, had vegetation such as rooted and un-rooted floating plants (e.g. *Hydrilla *spp, *Nelimbium *spp, *Salvinea *spp and *Eichornia *spp) and green algae. *An. subpictus *B/*An. sundaicus *larvae were also collected ~ 15 m from land in the shallow waters of a lagoon at Kallady where the salinity was 30 ppt. The larvae at this site were found associated with marine algae and plants and exposed to direct sunlight [Figure 2]. The pH and dissolved oxygen concentrations of the breeding sites where the larvae were collected varied from 7.1 to 8.4 and 1.1 to 2.6 mg/L respectively.

### Laboratory investigations on salinity tolerance of larvae

**Figure 3:**
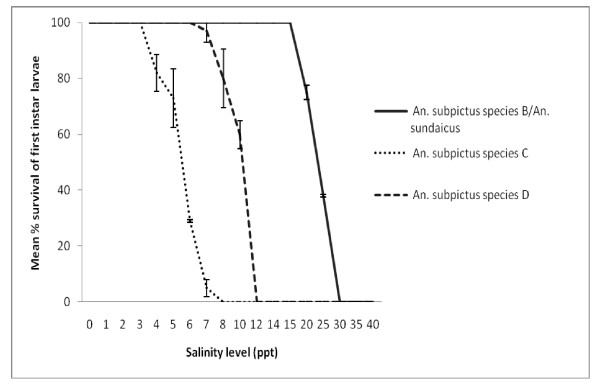
**Mean percentage survival to adulthood of first instar larvae of *An. subpictus *B/ *An. sundaicus*, and *An. subpictus *C and D at different levels of salinity**.

**Figure 4:**
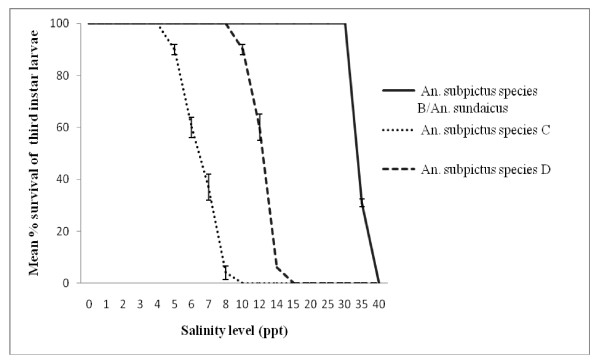
**Mean percentage survival to adulthood of third instar larvae of *An. subpictus *species B/*An. sundaicus*, and *An. subpictus *C and D at different levels of salinity**.

Larvae from mosquitoes with characteristics of *An. subpictus *B/*An. sundaicus*, and *An. subpictus *C and D differed in their ability to tolerate salinity. All first instar larvae with characteristics of *An. subpictus *B/*An. sundaicus *survived in water with up to 15 ppt salinity [Figure 3]. In contrast, 100% survival of larvae of *An. subpictus *C and D were only obtained at up to 3 ppt and 6 ppt salinity respectively. Also the third instar larvae of *An. subpictus *B/*An. sundaicus *recorded 100% survival at up to 30 ppt salinity whereas those classified as *An. subpictus *C and D showed 100% survival at only up to 4 ppt and 8 ppt salinity respectively [Figure 4].

## Discussion

Some members of the different anopheline species complexes show high salinity tolerance and are associated with coastal malaria transmission. *Anopheles melas *and *Anopheles merus *within the *Anopheles gambiae *complex are examples from Africa [17]. *Anopheles farauti s.s*. and *Anopheles irenicus *(formerly designated *An. farauti *No. 7) in the Farauti Complex are reported to be salinity-tolerant in Australasia [13,18]. Malaria vectors of the *An. sundaicus *complex in Southeast Asia are well known brackish water breeders that are also able to breed in freshwater [13]. *An. sundaicus *larvae (cytotype D) on the Car Nicorbar Island were collected from breeding sites with salinity ranging from 0 to 14 ppt [19]. In Indonesia, *An. sundaicus s.l *and *An. subpictus s.l *populations cohabit in the same breeding sites with salinity ranging from 5 to 10 ppt [20]. *An. subpictus *B/*An. sundaicus *were collected previously from stagnant brackish water bodies in Kallady and Oluvil in Eastern Sri Lanka [15]. The present findings show that *An. subpictus *B/*An. sundaicus *populations are present in breeding sites containing algae and exposed to sunlight with 0 to 30 ppt salinity in Sri Lanka. *An. sundaicus s.l*. and *An. subpictus s.l*. have also been reported by others to prefer breeding in sunlit sites that contain algae [20]. The pH and dissolved oxygen concentrations observed here fall within the ranges reported for *An. subpictus s.l*. and *An. sundaicus s.l*. larval breeding habitats elsewhere [19,20].

The collection of adult female mosquitoes and larvae of the different sibling species of the Subpictus Complex showed an association in inland and coastal areas. Sibling species A, C and D were generally predominant in inland localities even though their larvae were found to tolerate salinity levels up to 4 ppt in breeding sites. On the other hand, *An. subpictus *B/*An. sundaicus *tended to be more prevalent in coastal localities but their larvae were found to tolerate wide range of salinity levels (0 to 30 ppt). This shows that Sri Lankan *An. subpictus *B/*An. sundaicus *are similar to malaria vectors of the Sundaicus Complex in being able to tolerate a range of salinities and breed in fresh and brackish waters.

This is the first study to evaluate the salinity tolerance of the *An. subpictus*-like mosquitoes under both field and laboratory conditions. The greater salinity tolerance of *An. subpictus *B/*An. sundaicus *compared to *An. subpictus *C and D larvae observed in laboratory studies is consistent with their presence in more brackish water in nature. The coastal populations of the Subpictus Complex have been particularly incriminated as malaria vectors in India [21,22] and the Puttalam district in western Sri Lanka [11]. The ability of *An. subpictus *B/*An. sundaicus *to breed in brackish (0.5-30 ppt salt) and fresh water (<0.5 ppt salt) indicates that it is versatile enough to transmit malaria in large parts of Sri Lanka, which is a relatively small island with an extensive coastline and large tracts of brackish water bodies that extend deep inland. This report is also the first to show that *An. subpictus *B/*An. sundaicus *is able to breed in the shallow, open waters of a lagoon, the Kallady lagoon, in Sri Lanka. The Northern, Eastern and Northwestern provinces of Sri Lanka in particular have many lagoons, with similar characteristics to the Kallady lagoon, as well as other types of highly brackish water bodies [23] that offer potential breeding sites for *An. subpictus *B/*An. sundaicus*.

The use of morphological characteristics of eggs and larvae alone to differentiate members of the *An. subpictus *complex can have drawbacks [15]. DNA sequence-based tests suitable for field use are not available for differentiating *An. subpictus *sibling species and the reported cytogenetic differences between them are not useful for field studies [8]. Further studies to correlate the morphological, cytogenetic and molecular characteristics of members of the Subpictus Complex are clearly needed. However, salinity tolerance tests have been reported to be useful in separating morphologically similar members of anopheline species complexes. Sweeney [24] reported that exposure of the first instar larvae to sea water for 1 h would help separating *An. farauti *No. 1 (*An. farauti s.s*.) from other members of the Farauti Complex. Similarly, a salinity tolerance test, using 3 parts sea water having 3.2% NaCl and 1 part fresh water which is expected to be 24 ppt salinity, has been reported to separate species A and B of the Subpictus Complex in India [14]. The present study suggests that larval tolerance to 15 ppt salinity may provide a simple and inexpensive method for use in field studies to separate mosquitoes with morphological characteristics of *An. subpictus *B/*An. sundaicus *from others with characteristics of *An. subpictus *species C and D in Sri Lanka. Additional investigations are needed to determine the applicability of the proposed test to sibling species A in Sri Lanka, which is generally regarded as a freshwater breeding mosquito species.

The incidence of malaria in Sri Lanka, including in its Eastern and Northwestern provinces, has markedly decreased in the past decade due in part to an effective vector control program [1,2]. It has previously been proposed that rising sea levels due to global warming may increase the breeding of salinity-tolerant malaria vectors in coastal areas [25]. Taken together with the present findings, it may therefore be prudent to carefully monitor the breeding of malaria vectors in coastal brackish waters, and apply appropriate control measures where necessary, in order to maintain good malaria control in Sri Lanka. Similar considerations also apply to many Southeast Asian countries. Water management involving tidal flushing of breeding sites and source reduction using salinization are sometimes adopted to eliminate mosquito breeding [26,27]. However this approach in Sri Lanka and elsewhere in Southeast Asia needs careful consideration when vectors such as *An. subpictus *B/*An. sundaicus*, that are able to breed in brackish and saline waters, are present.

## Conclusions

The four sibling species A, B, C and D of *An. subpictus *identified through morphological characteristics attributed to Indian members of the *An. subpictus *complex, are present in Sri Lanka. *An. subpictus *A, C and D are generally predominant in inland areas, while *An. subpictus *B/*An. sundaicus *tends to be more prevalent in coastal localities. *An. subpictus *B/*An. sundaicus *in Sri Lanka is also able to breed in fresh to highly brackish waters including the Kallady lagoon with a salinity of 30 ppt. Brackish waters of similar high salt concentration are present in many coastal areas of Sri Lanka and Southeast Asian countries. This heightens the risk of malaria transmission in coastal areas which may be further exacerbated by rising sea levels increasing ground water salinization. Systematic monitoring of larval breeding habitats along the coastal belts of Sri Lanka and many Southeast Asian countries and the development of appropriate vector control measures are therefore needed. Based also on laboratory studies on salinity tolerance of larvae it is proposed that tolerance to 15 ppt salinity may constitute a simple method for differentiating *An. subpictus *B/*An. sundaicus *larvae from those of *An. subpictus *species C and D in field studies.

## Methods

### Mosquito collection and identification of sibling species of the Subpictus Complex

Adult anopheline mosquitoes were collected monthly in the two-year period July 2008 to June 2010 from five sites *viz*. Oluvil (coastal locality ~ 2 km from the coast) Chenkalady (inland locality ~ 5 km from the coast) and Muthur (inland locality ~ 3 km from the coast; coastal locality ~ 1 km from the coast) in the districts of Ampara, Batticaloa and Trincomalee respectively of the Eastern province and from Thonikkal (inland locality ~ 10 km from the coast) in the Puttalam district of the Northwestern province, all of which are located in the dry zone of Sri Lanka [Figure 1]. Collection sites located < 2.5 km from the coast are termed coastal sites. Cattle baited hut (CBHC) and cattle baited net (CBNC) collection techniques were used to collect adult female mosquitoes.

Larvae were also collected between February 2009 and June 2010 from coastal and inland locations in four administrative districts: Periakalappu (coastal locality ~ 1 km from the coast) and Dheegavavi (inland locality ~ 26 km from the coast) in the Ampara district, Thonikkal (inland locality ~ 10 km from the coast) in the Puttalam district, Chenkalady (inland locality ~5 km from the coast) and Kallady lagoon in the Batticaloa district and Muthur (coastal locality ~ 1 km from the coast) and Palaiyutru (inland locality ~2.5 km from the coast) in the Trincomalee district [Figure 1] using an 8 cm diameter and 240 ml capacity dipper as previously described [2]. Salinity of water samples was measured using a salinometer (Atago, Japan). The pH (Hanna Instruments, HI 98128, Rumania) and dissolved oxygen (Hanna Instruments, HI 8043, Rumania) were also measured in the collected water samples.

The collected adults and larvae were brought to the Zoology laboratory of the Eastern University and identified as *An. subpictus s.l*. using published keys [28-30]. Morphologically identified blood-fed females were maintained individually and single female F_1 _progenies were raised as described previously [31]. The sibling species status of females laying eggs was determined through the reported number of ridges in the floats of egg i.e. species A, 31-36; *An. subpictus *B/*An sundaicus*, 16-20; species C, 25-29 and species D, 21-24 [7]. Five to ten eggs from each female were placed on a clean microscopic slide and the number of ridges on floats counted under a light microscope (x4, Olympus). Larvae emerging from identified isofemale progenies were used for salinity tolerance experiments.

### Salinity tolerance tests on larvae of *An. subpictus *sibling species

*An. subpictus *B/*An. sundaicus, An. subpictus *C and *An. subpictus *D larvae were pooled separately. There were insufficient numbers of *An. subpictus *species A females in field collections to generate the required numbers of larvae for salinity tolerance tests. First and early third instar larvae of each sibling species were exposed to different salinity levels viz. 0, 1, 2, 3, 4, 5, 6, 7, 8, 10, 12, 14, 15, 20, 25, 30, 35, 40 ppt ( parts per thousand of salt). Required salinities were obtained either by adding tap water (0 ppt salinity) or NaCl to seawater (36 ppt salinity) [14,32]. Salinty was measured using a refractor salinometer (Atago, Japan). Twenty larvae in 150 ml capacity plastic containers containing 100 ml of water of specific salinity were maintained at room temperature (28 ± 2°C) until their emergence as adults. Three replicates with 20 larvae each were run in parallel at each salinity level. Plastic lids were used to cover the containers to minimise evaporation. Test media were changed every alternate day. Larvae were fed twice daily with locally available powered fish meal. The number of adults emerging was determined and the results recorded as the mean percentage survival of larvae to reach adulthood at each salinity level ± standard error of the mean.

## Competing interests

The authors declare that they have no competing interests.

## Authors' contributions

SNS and RR conceived the study. PJJ performed field collections. PJJ and SNS carried out laboratory studies. SNS and RR wrote the manuscript. All authors read and approved the final manuscript.
